# Haftung bei Suizid während stationärer Behandlung

**DOI:** 10.1007/s00115-025-01889-5

**Published:** 2025-09-01

**Authors:** Johannes Kornhuber, Antonia Stummvoll

**Affiliations:** 1 Psychiatrische und Psychotherapeutische Klinik, Friedrich-Alexander-Universität Erlangen-Nürnberg (FAU), Schwabachanlage 6, 91054 Erlangen, Deutschland; 2 Lehrstuhl für Öffentliches Recht IV, Universität Bayreuth, Universitätsstraße 30, 95447 Bayreuth, Deutschland

**Keywords:** Suizidprävention, Haftungsrecht, Psychiatrische Versorgung, Risikoeinschätzung, Klinisches Risikomanagement, Suicide prevention, Liability, legal, Psychiatric care, Risk assessment, Risk management, clinical

## Abstract

**Hintergrund:**

Suizide während stationärer Behandlung stellen für Kliniken und Behandelnde ein belastendes Ereignis dar und werfen Fragen nach haftungsrechtlichen Konsequenzen auf. Sie erfordern eine differenzierte Betrachtung im Spannungsfeld zwischen Patientenschutz und Therapienotwendigkeiten.

**Ziel der Arbeit:**

Analyse der langjährigen Rechtsprechung zur Identifikation praxisrelevanter Grundsätze für das klinische Risikomanagement bei Suizidalität.

**Material und Methoden:**

Systematische Auswertung von 22 höchst- und obergerichtlichen Entscheidungen deutscher Gerichte (1985–2025) zu Suiziden während stationärer Behandlung.

**Ergebnisse:**

Die Rechtsprechung erkennt die Unmöglichkeit einer absoluten Suizidprävention an und räumt dem psychiatrischen Behandlungsteam einen erheblichen Ermessensspielraum ein. Maßgeblich für die rechtliche Beurteilung ist die Ex-ante-Perspektive des Behandlers. Konkrete Sorgfaltspflichten ergeben sich bei akuter Suizidalität, Risikosituationen wie Lockerungen und bei erforderlichen baulichen und organisatorischen Maßnahmen. Die Abwägung zwischen Sicherungsinteresse und therapeutischen Erfordernissen steht im Mittelpunkt.

**Diskussion:**

Die Rechtsprechung zeigt ein differenziertes Verständnis für die klinischen Herausforderungen der Suizidprävention. Die identifizierten Bewertungsmaßstäbe bieten Orientierung für die Praxis und betonen die Notwendigkeit individueller Risikobewertungen sowie der Dokumentation aller relevanten Entscheidungen.

**Zusatzmaterial online:**

Zusätzliche Informationen sind in der Online-Version dieses Artikels (10.1007/s00115-025-01889-5) enthalten.

## Abstract (en)

**Background:**

Suicide during inpatient treatment represents distressing events for clinics and medical staff while raising questions about liability consequences. They require a differentiated approach balancing patient protection and therapeutic necessities.

**Objective:**

To analyze long-standing case law to identify practice-relevant principles for clinical risk management regarding suicidal ideation.

**Material and methods:**

Systematic evaluation of 22 decisions from German high and supreme courts (1985–2025) concerning suicides during inpatient treatment.

**Results:**

Case law acknowledges the impossibility of absolute suicide prevention and grants psychiatric treatment teams considerable discretionary latitude. The legal assessment is based on the treating physician’s ex-ante perspective. Specific duties of care arise in cases of acute suicidal ideation, risk situations such as therapeutic relaxations and necessary structural and organizational measures. The balance between security interests and therapeutic requirements remains central.

**Conclusion:**

The jurisprudence demonstrates a nuanced understanding of the clinical challenges in suicide prevention. The identified assessment standards provide orientation for the clinical practice and emphasize the necessity of individual risk assessments and documentation of all relevant decisions.

## Hinführung zum Thema

Suizide während stationärer Behandlung stellen Behandlungsteams vor Entscheidungen unter Unsicherheit: Wie wird akute Eigengefährdung eingeschätzt, welches Sicherungsniveau ist erforderlich und wann sind Lockerungen vertretbar? Die Dynamik psychiatrischer Verläufe, schwankende Einwilligungsfähigkeit und begrenzte Vorhersagbarkeit mindern die Entscheidungssicherheit. Strukturierte Risikoeinschätzung, klare Zuständigkeiten und nachvollziehbare Dokumentation sind daher zentral, ebenso klinikinterne Standards für Krisensituationen. Vor diesem Hintergrund kommt dem rechtlichen Rahmen eine besondere Orientierungsfunktion zu.

## Einleitung

Suizide während stationärer Behandlung stellen für medizinische Einrichtungen und behandelnde Ärzte ein besonders belastendes Ereignis dar. Neben der persönlichen Betroffenheit der Angehörigen und des Behandlungsteams werfen solche Fälle stets die Frage nach möglichen haftungsrechtlichen Konsequenzen auf.

Die vorliegende Arbeit untersucht die langjährige Rechtsprechung zu diesen Fragen mit dem Ziel, praxisrelevante Grundsätze für das klinische Risikomanagement zu identifizieren. Der Fokus liegt dabei auf der Analyse, wie Gerichte die medizinischen Sachverhalte bewerten und welche rechtlichen Maßstäbe sie an das ärztliche Handeln anlegen.

Dabei werden hier ausschließlich Fälle untersucht, in denen ein Suizid verhindert werden soll, weil er nicht frei-verantwortlich stattfindet.

## Rechtliche Einordnung

Kommt ein Patient aufgrund einer Pflichtverletzung des medizinischen Personals durch einen Suizidversuch zu Tode oder wird verletzt, drohen verschiedene rechtliche Konsequenzen.

In den meisten hier betrachteten Entscheidungen wurde auf *zivilrechtlichem Weg* Schadensersatz von den Verantwortlichen gefordert. Geltend gemacht werden können diese Ansprüche vom überlebenden, verletzten Patienten selbst oder bei erfolgtem Suizid von dessen hinterbliebenen Angehörigen, die neben Schmerzensgeld (siehe etwa [4]) beispielsweise auch den durch die Tötung ausfallenden Unterhalt geltend machen können. Auf ein Mitverschulden des Patienten gem. § 254 Bürgerliches Gesetzbuch (BGB), das die Anspruchshöhe reduzieren könnte, kann sich im Fall des nichtfreiverantwortlichen Suizids nicht berufen werden [3, 5].

Denkbar ist außerdem eine *strafrechtliche Verfolgung*. Für Personen, die Garanten sind, etwa weil sie als Ärzte oder Pflegepersonal die Behandlung/Betreuung des Patienten übernommen haben [2], kommt eine Strafbarkeit durch Unterlassen in Betracht. Wenn kein Vorsatz vorliegt, aber der Garant die ihm obliegenden Sorgfaltspflichten gegenüber dem Patienten fahrlässig nicht beachtet hat, kommt eine fahrlässige Tötung (§ 222 Strafgesetzbuch, StGB) durch Unterlassen in Betracht [1].

Für Ärzte sind ferner unter Umständen *berufsrechtliche Konsequenzen* denkbar.

Sozialversicherungsträger können Verantwortliche in *Regress* nehmen, wenn Folgebehandlungen oder Behinderungen des Patienten durch den Suizidversuch entstehen.

## Methodik

Für die vorliegende Analyse wurden 22 höchst- und obergerichtliche Entscheidungen deutscher Gerichte aus dem Zeitraum 1985 bis 2025 herangezogen, die sich mit Suiziden während stationärer Krankenhausbehandlung befassen (siehe Anhang). Die Fälle betrafen überwiegend die stationäre psychiatrische Krankenhausbehandlung von Erwachsenen; Suizide in internistischen oder allgemeinmedizinischen Settings wurden jedoch ebenfalls berücksichtigt. Ein Urteil bezieht sich auf die Behandlung einer 17-jährigen Patientin in einer Klinik für Kinder- und Jugendpsychiatrie. Ein Urteil bezieht sich auf den Suizid eines Patienten am Abend des Entlasstags, formal also kurz nach der Entlassung. Die untersuchten Suizidfälle haben nicht zwingend in den Räumlichkeiten der psychiatrischen Klinik stattgefunden, sondern teilweise auch außerhalb, beispielsweise bei Ausgängen oder Beurlaubungen.

Der Fokus lag auf den rechtlichen Bewertungsmaßstäben, die von den Gerichten bei der Beurteilung von behaupteten Behandlungsfehlern im Zusammenhang mit Suiziden angelegt wurden. Es wurden nur veröffentlichte Gerichtsurteile mit detailreichen Informationen ausgewertet. Dabei ist zu beachten, dass nur ein kleiner Teil der Gerichtsurteile in juristischen Datenbanken veröffentlicht wird. Vor einer Publikation müssen Urteile anonymisiert werden, was insbesondere bei komplexen medizinrechtlichen Sachverhalten einen erheblichen Aufwand darstellt. Dies führt zu einer systematischen Selektionsverzerrung, da vor allem höherinstanzliche und grundsätzliche Entscheidungen veröffentlicht werden, während viele erstinstanzliche Urteile und außergerichtliche Einigungen nicht zugänglich sind.

Die extrahierten Daten wurden nach thematischen Schwerpunkten kategorisiert und systematisch aufbereitet. Für jede identifizierte Entscheidung wurde ein praxisrelevantes Ergebnis formuliert, das die wesentliche rechtliche Aussage zusammenfasst. Die Synthese der Ergebnisse erfolgte durch die Ableitung allgemeiner Grundsätze aus den einzelnen Entscheidungen.

## Grundlegendes Urteil des Bundesgerichtshofs

Die vom Bundesgerichtshof (BGH) (23.09.1993, III ZR 107/92) formulierten Grundsätze finden sich in allen nachfolgenden Urteilen wieder und bilden die Basis für die rechtliche Beurteilung von Suiziden während stationärer Behandlung. Sie werden in der Rechtsprechung kontinuierlich fortentwickelt und an die spezifischen Fallkonstellationen angepasst.

„Ein Suizid während des Aufenthalts in einem Psychiatrischen Krankenhaus kann niemals mit absoluter Sicherheit vermieden werden, gleich, ob die Behandlung auf einer offenen oder auf einer geschlossenen Station unter Beachtung aller realisierbaren Überwachungsmöglichkeiten durchgeführt wird. Eine lückenlose Überwachung und Sicherung, die jede noch so fernliegende Gefahrenquelle ausschalten könnte, erscheint nicht denkbar. Zudem sind stets die Erfordernisse der Medizin zu beachten, die nach moderner Auffassung gerade bei psychisch Kranken eine vertrauensvolle Beziehung und Zusammenarbeit zwischen Patient und Arzt sowie Krankenhauspersonal auch aus therapeutischen Gründen als angezeigt erscheinen lassen. Entwürdigende Überwachungs- und Sicherungsmaßnahmen, soweit sie überhaupt zulässig sind, können nach heutiger medizinischer Erkenntnis eine erfolgversprechende Therapie gefährden. Ein zum Selbstmord Entschlossener findet ohnehin Mittel und Wege, seinen Plan auszuführen.“

## Juristische Bewertungsmaßstäbe

### Grundlegende Rechtsprinzipien

#### Rechtliche Sorgfaltspflichten

##### Beschreibung.

Umfasst die grundlegenden rechtlichen Verpflichtungen von Kliniken und medizinischem Personal, suizidgefährdete Patienten zu schützen. Beinhaltet sowohl die allgemeine Pflicht zur Verhinderung von Selbstgefährdungen als auch die Grenzen dieser Pflicht.

##### Schlussfolgerungen.

Die Rechtsprechung betont durchgängig die Pflicht von Behandlern, suizidgefährdete Patienten zu schützen, jedoch immer im Rahmen des Erforderlichen und Zumutbaren. Die Schutzpflicht ist nicht absolut, sondern unterliegt einer Abwägung mit therapeutischen Erfordernissen und der Patientenautonomie. Insbesondere ist eine dauerhafte Überwachung des Patienten nicht geboten, die das Therapieziel durch eine Störung des Vertrauensverhältnisses zwischen Arzt und Patient gefährden kann.

##### Ankerbeispiele.

- „Aufgrund der allgemeinen Verkehrssicherungspflicht ist der Betreiber einer psychiatrischen Klinik verpflichtet, dafür Sorge zu tragen, dass die Patienten nicht vermeidbaren Gefahren und Selbstgefährdungen ausgesetzt sind“ (Oberlandesgericht [OLG] Zweibrücken, Urteil vom 22.12.2009 – 5 U 5/07).
- „Diese Pflicht besteht allerdings, wie das Berufungsgericht nicht verkannt hat, nur in den Grenzen des Erforderlichen und des für das Krankenhauspersonal und die Patientin Zumutbaren“ (BGH, Urteil vom 23.09.1993 – III ZR 107/92).

#### Kausalitätsnachweis

##### Beschreibung.

Umfasst die Anforderungen an den Beweis des Zusammenhangs zwischen einer Pflichtverletzung und dem eingetretenen Suizid, insbesondere der Frage, ob der Suizid durch pflichtgemäße Behandlung hätte verhindert werden können.

##### Schlussfolgerungen.

Die Gerichte stellen hohe Anforderungen an den Beweis der Kausalität zwischen einer Pflichtverletzung und dem Suizid. Der Geschädigte muss beweisen, dass der Suizid bei pflichtgemäßem Handeln mit hinreichender Wahrscheinlichkeit verhindert worden wäre.

##### Ankerbeispiel.

- „Begeht der Patient während des unbeaufsichtigten Duschens Suizid, haftet der Träger des Krankenhauses nur, wenn der Behandlungsfehler der Pflegekraft ursächlich für den Suizid gewesen ist, also wenn mit der erforderlichen Gewissheit feststeht, dass der Patient nicht verstorben wäre, wenn zuvor eine psychiatrische Exploration durch einen Arzt stattgefunden hätte. Die Beweislast dafür tragen die Anspruchsteller“ (OLG Köln, Urteil vom 21.08.2024 – I‑5 U 127/23).

#### Grenzen der Suizidprävention

##### Beschreibung.

Bezieht sich auf die prinzipielle Nichtvorhersehbarkeit und Nichtvermeidbarkeit von Suiziden trotz angemessener Sicherungsmaßnahmen.

##### Schlussfolgerungen.

Die Gerichte erkennen an, dass ein Suizid selbst bei Anwendung aller zumutbaren Sicherungsmaßnahmen nicht mit absoluter Sicherheit verhindert werden kann. Wer fest entschlossen ist, sich das Leben zu nehmen, findet erfahrungsgemäß eine Möglichkeit, dies umzusetzen.

##### Ankerbeispiel.

- „Restzweifel über das Fortbestehen einer Suizidalität des Patienten können in der psychiatrischen Behandlung nicht ausgeräumt werden. Dazu müsste der Arzt nicht nur die Gedanken des Patienten lesen, sondern diese auch, was nicht einmal dem Patienten selbst möglich ist, antizipieren können. Eine sichere Suizidprophylaxe ist deshalb bei Wahrung der Menschenwürde des Patienten und eines zeitgemäßen Therapiekonzeptes nicht möglich“ (OLG München, Urteil vom 13.01.2011 – 1 U 4927/09).

#### Abwägung zwischen Therapiezielerreichung und Sicherung

##### Beschreibung.

Umfasst die Balance zwischen der für das Erreichen des Therapieziels notwendigen Selbstbestimmung des Patienten und der Notwendigkeit von Schutzmaßnahmen zur Suizidprävention.

##### Schlussfolgerungen.

Die Gerichte betonen, dass bei psychiatrischen Behandlungen ein Spannungsverhältnis zwischen der Therapiezielerreichung und notwendigen Sicherungsmaßnahmen besteht. Diese Abwägung muss im Einzelfall erfolgen und berücksichtigen, dass zu strikte Verwahrung therapeutisch kontraproduktiv sein kann.

##### Ankerbeispiel.

- „… die Inkaufnahme von Risiken – auch des Risikos der Selbstschädigung – therapeutisch notwendig sein kann. Der Psychiater hat über die Gefahr einer schrittweise zu gewährenden Freiheit zu entscheiden. Gewährt er diese Freiheit nicht, wird er zwar eher vor dem größten therapeutischen Fehlschlag, dem Tod des Patienten, bewahrt, verhindert aber gleichzeitig die zur Lebensbejahung notwendige Verwirklichung von Autonomie“ (OLG Naumburg, Urteil vom 08.02.2000 – 1 U140/99).

### Beurteilung der ärztlichen Sorgfalt

#### Therapeutische Ermessensspielräume

##### Beschreibung.

Bezieht sich auf den Beurteilungs- und Entscheidungsspielraum von Ärzten bei der Behandlung suizidgefährdeter Patienten, insbesondere bei der Abwägung zwischen therapeutischen Zielen und Sicherheitsaspekten.

##### Schlussfolgerungen.

Die Gerichte erkennen durchgängig an, dass Ärzte bei der Behandlung suizidgefährdeter Patienten einen erheblichen Ermessensspielraum haben. Die Inkaufnahme von Risiken kann therapeutisch notwendig sein, um die Eigenverantwortlichkeit der Patienten zu stärken und eine erfolgversprechende Therapie zu ermöglichen.

##### Ankerbeispiel.

- „Der Arzt ist im Rahmen der verfassungsrechtlich garantierten Therapiefreiheit berechtigt und verpflichtet, die ihm geeignet erscheinende diagnostische und therapeutische Behandlungsmethode auszuwählen. Gerade im Bereich psychiatrischer Behandlungen ist dem Arzt ein weiter diagnostischer und therapeutischer Beurteilungs- und Ermessensspielraums zuzubilligen“ (Landesberufsgericht für Heilberufe Münster, Beschluss vom 20.04.2016 – 6t E 928/14.T).

#### Ex-ante- vs. Ex-post-Betrachtung

##### Beschreibung.

Bezieht sich auf den maßgeblichen Zeitpunkt der Beurteilung von ärztlichen Entscheidungen – ob diese aus der Perspektive des Behandlungszeitpunkts (ex ante) oder aus der Perspektive nach dem Schadenseintritt (ex post) zu bewerten sind.

##### Schlussfolgerungen.

Die Gerichte betonen durchgängig, dass ärztliche Entscheidungen aus der Ex-ante-Perspektive zu beurteilen sind, also aus Sicht der Behandler zum Zeitpunkt der Behandlung. Eine nachträgliche Beurteilung aufgrund des eingetretenen Schadens ist nicht zulässig.

##### Ankerbeispiel.

- „Es gibt kaum einen Suizid in der Psychiatrie, der sich aus der ex post Perspektive nicht hätte verhindern lassen. Man darf daraus aber nicht den Schluss ziehen, dass dieser ex ante hätte verhindert werden können und müssen“ (OLG München, Beschluss vom 13.01.2011 – 1 U 4927/09).

#### Methodik der Suizidalitätseinschätzung

##### Beschreibung.

Umfasst die fachlichen Kriterien und methodischen Vorgehensweisen zur Beurteilung der Suizidgefahr bei Patienten.

##### Schlussfolgerungen.

Die Gerichte betonen die Notwendigkeit einer sorgfältigen Exploration und Beurteilung der Suizidalität. Dabei werden verschiedene Kriterien wie Krankheitsbild, Vorgeschichte, aktuelle Äußerungen und Verhaltensweisen des Patienten berücksichtigt.

##### Ankerbeispiel.

- „Bei einem Patienten mit akuter Suizidalität, bei dem sowohl eigene Suizidgedanken als auch das Hören ‚suizidimperative Stimmen‘ im Raum stehen, bedarf es einer fundierten Evaluation, bevor man als Behandler von einer Entaktualisierung des Geschehens ausgehen kann“ (OLG Hamm – 20.12.2022 – 26 U 15/22).

#### Dokumentationsanforderungen

##### Beschreibung.

Umfasst die rechtlich geforderten Inhalte und die Qualität der klinischen Dokumentation bei der Behandlung suizidgefährdeter Patienten.

##### Schlussfolgerungen.

Die Gerichte betonen die Bedeutung einer angemessenen Dokumentation der Behandlung, insbesondere der Einschätzung der Suizidalität und der getroffenen Sicherungsmaßnahmen. Eine unzureichende Dokumentation kann zu Beweiserleichterungen für den Kläger führen.

##### Ankerbeispiel.

- „Obwohl diese nicht vordringlich der forensischen Beweissicherung dient, wird ihr doch im Hinblick auf die aus medizinischer Sicht wesentlichen medizinischen Fakten, die für die ärztliche Diagnose und Therapie von Bedeutung und daher aufzeichnungspflichtig sind, nach gefestigter Rechtsprechung eine Indizwirkung im Hinblick auf ihre Richtigkeit und Vollständigkeit zugeschrieben. Diese bezieht sich gleichermaßen in positiver Hinsicht darauf, dass die dokumentierten Befunde erhoben und die dokumentierten Behandlungsmaßnahmen ergriffen wurden […], wie in negativer Hinsicht darauf, dass nicht erwähnte dokumentationspflichtige Befunde nicht erhoben und nicht dokumentierte Behandlungsmaßnahmen nicht ergriffen wurden“ (OLG Köln, Urteil vom 17.06.2024 – 5 U 112/23).

#### Häufigkeit der Sichtung der Patienten bei Suizidalität

##### Beschreibung.

Beschreibt die erforderliche Frequenz der Kontrolle und Überwachung suizidgefährdeter Patienten.

##### Schlussfolgerungen.

Die Gerichte betonen, dass die Häufigkeit der Sichtung und Überwachung von der individuellen Suizidgefahr abhängt. Bei akuter Suizidgefahr kann eine engmaschige bis ständige Überwachung erforderlich sein.

##### Ankerbeispiel.

- „Der Facharztstandard sieht für Hochrisikopatienten wie den Kläger eine kontinuierliche, regelmäßige – bei Fortbestehen der akuten Suizidalität tägliche – Befundung des psychischen Erlebens und der Gemütsverfassung vor, um die Entwicklung im Verlauf beurteilen zu können und eine sichere Grundlage für eine Diagnoseänderung und für erforderliche Therapiemaßnahmen zu schaffen“ (OLG Hamm – 20.12.2022 – 26 U 15/22).

### Konkrete Sicherungsmaßnahmen

#### Bauliche, technische und personelle Sicherungsmaßnahmen

##### Beschreibung.

Umfasst bauliche, technische und personelle Maßnahmen zur Verhinderung von Suiziden in psychiatrischen Einrichtungen.

##### Schlussfolgerungen.

Die Gerichte stellen fest, dass bauliche und technische Sicherungsmaßnahmen notwendig sind, jedoch nicht absolut sein müssen. Die erforderlichen Maßnahmen hängen vom Einzelfall und der konkreten Suizidgefahr ab.

##### Ankerbeispiel.

- „Auch geschlossene psychiatrische Stationen dienen in erster Linie therapeutischen Zwecken; sie sind keine Hochsicherheitstrakte. Patienten wünschen, zum Beispiel um frische Luft erhalten zu können, kippbare Fenster, was auf Grund der mechanischen Gegebenheiten für handwerklich begabte Patienten bereits beste Möglichkeiten bietet, die Kippmechanismen auszuhebeln und die Fenster entsprechend zu öffnen. Auch die Vergitterung von Fenstern, in Gefängnissen üblich, scheidet bei einer Klinik aus vielfältigen Gründen aus. Ein Entweichen von einer geschlossenen Station ist daher in der Regel nicht durch bauliche Maßnahmen komplett zu verhindern, wenn man den Charakter einer Klinik, die in erster Linie zur Behandlung seelisch kranker Menschen gedacht ist, nicht zerstören will“ (OLG Zweibrücken – Urteil vom 22.12.2009 – 5 U 5/07).

#### Latente Fluchtgefahr bei Suizidalität

##### Beschreibung.

Umfasst die rechtliche Pflicht, bei allen suizidgefährdeten Patienten – auch solchen ohne erkennbare akute Fluchttendenzen oder Gewalttendenz – mit Fluchtversuchen zur Verwirklichung von Selbstschädigungsabsichten zu rechnen und entsprechende organisatorische Vorkehrungen zu treffen.

##### Schlussfolgerungen.

Die Gerichte betonen, dass auch bei suizidgefährdeten Patienten ohne erkennbare akute Fluchttendenzen mit spontanen Fluchtversuchen gerechnet werden muss. Die Sicherheitsvorkehrungen müssen über individuelle Überwachungsmaßnahmen hinausgehen und strukturelle Lücken im Sicherungssystem schließen. Entscheidend ist nicht das individuelle Verhalten des Personals im Einzelfall, sondern die organisatorisch vorgegebenen Regeln und Strukturen. Diese Organisationspflicht besteht unabhängig von therapeutischen Erwägungen, wenn die Sicherungsmaßnahme das Therapiekonzept nicht beeinträchtigt.

##### Ankerbeispiel.

- „Bei suizidgefährdeten Patienten, auch solchen ohne erkennbare akute Fluchttendenz und insbesondere ohne Tendenz zur Flucht unter Gewaltanwendung, mußte mit der Möglichkeit gerechnet werden, daß sie zur Verwirklichung ihrer Selbstschädigungsabsichten versuchen könnten, durch die Tür zum Hof nach draußen zu gelangen, wenn die Tür nur von einer Person benutzt wurde und sich sonst niemand in dem Flur aufhielt, der den Fluchtversuch bemerken und verhindern konnte“ (OLG Düsseldorf, Urteil vom 10.01.1994 – 8 U 26/92).

### Besondere Risikosituationen

#### Risikokonstellationen bei Lockerungen

##### Beschreibung.

Umfasst besondere Gefahrensituationen und erhöhte Sorgfaltsanforderungen bei Lockerungsmaßnahmen wie Ausgang, Beurlaubungen oder Verlegung auf die offene Station.

##### Schlussfolgerungen.

Die Gerichte stellen erhöhte Anforderungen an die Sorgfalt bei Lockerungsmaßnahmen für suizidgefährdete Patienten. Die Entscheidung muss auf einer sorgfältigen Risikoabwägung basieren und besondere Vorsichtsmaßnahmen können erforderlich sein.

##### Ankerbeispiel.

- „Vor diesem Hintergrund war es unabdingbar, vor der Gestattung des Ausgangs mit Angehörigen am ##.01.2018 den bei der Patientin bestehenden Handlungsdruck durch erfahrenes Personal sorgfältig zu prüfen, idealerweise im Beisein des Angehörigen, mit welchem der Ausgang stattfinden soll“ (Landgericht (LG) Münster, Urteil vom 28.10.2021 – 111 O 75/19).

#### Angehörigeneinbeziehung

##### Beschreibung.

Umfasst die rechtliche Bedeutung der Information und Einbindung von Familienangehörigen bei der Suizidprävention, insbesondere bei Ausgängen und Beurlaubungen.

##### Schlussfolgerungen.

Die Gerichte betonen die Wichtigkeit der Information und Einbeziehung von Angehörigen bei der Betreuung suizidgefährdeter Patienten, insbesondere bei Ausgang oder Beurlaubungen. Die Verweigerung einer bestimmten Information kann einen Behandlungsfehler darstellen.

##### Ankerbeispiel.

- „Es wäre notwendig gewesen, die Person, mit welcher der Patientin der Angehörigenausgang gestattet wird, über die akute Suizidalität zu informieren. Dies ist hier unstreitig nicht geschehen“ (LG Münster, Urteil vom 28.10.2021 – 111 O 75/19).

#### Voraussetzungen für Zwangsmaßnahmen

##### Beschreibung.

Umfasst die rechtlichen Voraussetzungen für freiheitsentziehende oder freiheitsbeschränkende Maßnahmen bei suizidgefährdeten Patienten.

##### Schlussfolgerungen.

Die Gerichte betonen, dass Zwangsmaßnahmen nur als letztes Mittel in Betracht kommen und einer gesetzlichen Grundlage bedürfen. Sie sind nur zulässig, wenn eine akute Suizidgefahr besteht und mildere Mittel nicht ausreichen.

##### Ankerbeispiel.

- „Den behandelnden Ärzten hätte es am 29. Dezember 2018 oblegen, den Kläger von der Zurückstellung seines Beurlaubungswunsches zu überzeugen und – bei Erfolglosigkeit – die vorübergehende geschlossene Unterbringung nach dem PsychKG zu beantragen“ (OLG Hamm, – 20.12.2022 – 26 U 15/22).

### Verantwortungsverteilung

#### Verantwortungsverteilung im Behandlungsteam

##### Beschreibung.

Bezieht sich auf die Kompetenzen, Verantwortlichkeiten und Pflichten der verschiedenen Berufsgruppen im Behandlungsteam, insbesondere im Verhältnis zwischen Ärzten und Pflegekräften.

##### Schlussfolgerungen.

Die Gerichte erkennen an, dass auch Pflegekräfte in psychiatrischen Einrichtungen eine wichtige Rolle bei der Einschätzung der Suizidalität spielen können. Sie haben in bestimmten Grenzen die Kompetenz, eigenständige Entscheidungen zu treffen, und ärztliche Unterstützung hinzuziehen, wenn diese erforderlich ist.

##### Ankerbeispiel.

- „Es gehört zu den Aufgaben und zum Alltag von berufserfahrenen Pflegekräften in einer psychiatrischen Klinik, eine mögliche Suizidalität eines Patienten zu hinterfragen und zu beurteilen“ (OLG Köln, Urteil vom 21.08.2024 – I‑5 U 127/23).

## Verfahrensbeteiligte

Die Mehrheit der analysierten Fälle behandelt zivilrechtliche Verfahren, in denen in der Regel ein Anspruch auf Schadensersatz geltend gemacht wird. Die Kläger sind hierbei häufig Angehörige (Ehegatten, Kinder, Eltern) der durch Suizid verstorbenen Patienten. In einigen Fällen treten jedoch auch andere Beteiligte als Kläger auf. Die Beklagten sind in fast allen Fällen Krankenhausträger, die behandelnden Kliniken oder die direkt behandelnden Ärzte/Therapeuten.

## Entscheidungsausgang und Gründe

In nur fünf der 22 analysierten Fällen folgte das Gericht der Verurteilung der Beklagten bzw. hielt diese aufrecht. In diesen Fällen waren folgende Gründe ausschlaggebend.

### *Schuldhafter Organisationsmangel*.

Fehlende Anweisung, dass die Tür zum Hof nur unter Anwesenheit einer zweiten sichernden Person benutzt werden durfte (10.01.1994 – OLG Düsseldorf 8 U 26/92).

### *Konkrete bauliche Sicherungspflicht*.

Fenster müssen auch gegen Körperkraft gesichert sein, damit Patienten nicht hinausspringen können. Unzureichende Fenstersicherung, die gewaltsames Öffnen ermöglichte (14.02.2003 – OLG Hamburg 1 U 186/00).

*Anmerkung: Das nachfolgende Urteil des OLG Zweibrücken vom 22.12.2009 – 5 U 5/07 relativiert dies jedoch.*

*Vernachlässigung der Sicherungspflichten trotz fortbestehender Suizidalität. *Die Patientin hatte zuvor bereits zwei Suizidversuche in der Klinik unternommen, ohne dass eine signifikante Änderung der Risikofaktoren eingetreten war. Dem Risiko hätte durch Verbot eines eigenen Feuerzeugs unschwer begegnet werden können. Im Spannungsverhältnis zwischen Sicherungsinteresse und therapeutischen Lockerungen ist eine restriktive Handhabung geboten, wenn die konkrete Einschränkung (hier: kein eigenes Feuerzeug) das Therapiekonzept nicht gefährdet (03.03.2008 – OLG Koblenz 5 U 1343/07).

*Versäumnis, die Person, mit der der Patientin der Angehörigenausgang gestattet wurde, über die akute Suizidalität zu informieren *(2021.10.28 – LG Münster 111 O 75/19).

*Unzureichende Befunderhebung und vorschnelle Aufgabe der Annahme einer Suizidalität* (2022.12.20 – OLG Hamm 26 U 15/22).

## Schlussfolgerungen

Die Analyse der Gerichtsurteile zeigt eine differenzierte rechtliche Bewertung des Umgangs mit Suizidalität in medizinischen Einrichtungen. Die Rechtsprechung erkennt die Komplexität des Themas an und vermeidet pauschale Regelungen zugunsten einzelfallbezogener Abwägungen.

Das Spannungsfeld zwischen den Sicherungspflichten und den therapeutischen Erfordernissen wird durchgängig berücksichtigt. Die Gerichte räumen dem ärztlichen Personal einen weiten Ermessensspielraum ein und betonen, dass ein Suizid niemals mit absoluter Sicherheit verhindert werden kann. Gleichzeitig werden klare Sorgfaltspflichten definiert, besonders wenn eine akute Suizidgefahr erkennbar ist.

Nicht was im Nachhinein als richtig erscheint, sondern was zum Zeitpunkt der Behandlung erkennbar und geboten war, ist maßgeblich für die Beurteilung ärztlichen Handelns im Umgang mit suizidgefährdeten Patienten.

Eine sorgfältige Dokumentation ist zentral zur Haftungsvermeidung. Sie muss Befunde zur Suizidalität und die Entscheidungsgründe – etwa zu Lockerungen und zum Sicherungsniveau – nachvollziehbar festhalten und das Spannungsfeld zwischen Sicherung und therapeutischen Erfordernissen sichtbar machen. Klinikinterne Richtlinien zum Umgang mit suizidalen Patienten sollten regelmäßig aktualisiert werden. Die Kenntnis einschlägiger Rechtsprechung unterstützt die Optimierung der Organisation, erhöht die Behandlungsqualität und kann Suizidrisiken in psychiatrischen Einrichtungen reduzieren.

## Fazit für die Praxis

- Eine absolute Suizidprävention ist unmöglich – therapeutische Notwendigkeiten und das Gebot der Wahrung der Patientenwürde setzen der Überwachung Grenzen.
- Die Rechtsprechung bemisst ärztliches Handeln anhand der Ex-ante-Perspektive, nicht nach dem tatsächlichen Ausgang.
- Therapiekonzepte mit kontrollierten Risiken sind rechtlich anerkannt, sollten aber sorgfältig dokumentiert werden.
- Bei akuter Suizidalität sind intensivere Sicherungsmaßnahmen erforderlich, bei Lockerungen ist besondere Sorgfalt geboten.
- In kritischen Situationen (Ausgänge, Beurlaubungen) sollten Angehörige über Suizidalität informiert werden.
- Die sorgfältige Dokumentation der Suizidrisikoeinschätzung und getroffener Entscheidungen ist haftungsrechtlich essenziell.

## Supplementary Information

Anhang

## Data Availability

Alle Daten sind in diesem Manuskript enthalten; daneben gibt es keine Daten, die zur Verfügung gestellt werden könnten.
