# Supplementary material for: Liability in cases of suicide during inpatient treatment
Source: Nervenarzt. 2025 Sep 1;97(4):370–6. [Article in German] doi: 10.1007/s00115-025-01889-5 (PMC13315102; doi:10.1007/s00115-025-01889-5)
Supplement: Supplementary file 1 — Anhang [file 115_2025_1889_MOESM1_ESM.docx]

## Anhang

### BGH, Urteil vom 08.10.1985 - VI ZR 114/84

**Inhalt:** Bei der stationären Behandlung eines suizidgefährdeten Patienten kann der Krankenhausträger dem Patienten kein Mitverschulden entgegenhalten, wenn dieser trotz Behandlung einen Selbsttötungsversuch unternimmt.

**Sachverhalt:** Ein Patient befand sich in der neurologisch-psychiatrischen Abteilung eines Krankenhauses zur Behandlung nach LSD-Einnahme. Er stürzte sich über die Balustrade des Treppenhauses im zweiten Stock und zog sich erhebliche Verletzungen zu (Suizidversuch).

**Setting:** Neurologisch-psychiatrische Abteilung eines Krankenhauses (keine explizite Angabe, ob offene oder geschlossene Station)

**Verfahrensbeteiligte:** Kläger: Ersatzkasse (aus übergegangenem Recht eines Versicherten); Beklagter: Land (als Träger des Krankenhauses)

**Entscheidung:** Die Revision wurde zur erneuten Verhandlung zurückverwiesen. Der Krankenhausträger kann dem Patienten kein Mitverschulden entgegenhalten, da der Behandlungsauftrag gerade den Schutz vor selbstschädigenden Handlungen umfasst.

### BGH, Urteil vom 23.09.1993 - III ZR 107/92

**Inhalt:** Die Sorgfaltspflichten gegenüber suizidgefährdeten Patienten in einem psychiatrischen Krankenhaus sind begrenzt auf das Erforderliche und Zumutbare und müssen gegen Gesichtspunkte einer Therapiegefährdung durch allzu strikte Verwahrung abgewogen werden. Relevant ist die Ex-ante-Sicht. Die Beurteilung durch das Gericht bedarf regelmäßig eines sachverständigen Gutachters.

**Sachverhalt:** Eine Patientin hatte in einem psychiatrischen Krankenhaus mit einem Feuerzeug ihre Kleidung angezündet und erhebliche Verletzungen erlitten (Suizidversuch).

**Setting:** Psychiatrisches Krankenhaus (keine explizite Angabe, ob offene oder geschlossene Station)

**Verfahrensbeteiligte:** Kläger: Allgemeine Ortskrankenkasse; Beklagter: Landeswohlfahrtsverband Hessen (als Krankenhausträger)

**Entscheidung:** Der BGH hob das Berufungsurteil auf und verwies zurück, da nicht ausreichend geprüft wurde, ob nach Ex-ante-Sicht eine Selbstverbrennungsgefahr zu erkennen war und welche Schutzmaßnahmen geboten waren. Die Sicherungspflicht ist gegen Therapiegefährdung durch strikte Verwahrung abzuwägen. Die Beurteilung durch das Gericht bedarf regelmäßig eines sachverständigen Gutachters.

### OLG Düsseldorf, Urteil vom 10.01.1994 – 8 U 26/92

**Inhalt:** Psychiatrische Kliniken müssen durch organisatorische Maßnahmen sicherstellen, dass Ausgangstüren bei Benutzung durch Personal so abgesichert sind, dass suizidgefährdete Patienten nicht entweichen können.

**Sachverhalt:** Die Klägerin wurde nach einem Suizidversuch wegen fortbestehender Suizidgefahr in ein Psychiatrisches Fachkrankenhaus verlegt. Dort versuchte sie, sich mit einer Schere zu verletzen und wollte aus dem Fenster springen. Nach zweieinhalb Stunden Fixierung wurde sie auf eine andere Station verlegt. Am Abend gelang ihr die Flucht durch eine Tür zum Hof, die ein Pfleger zum Verlassen des Gebäudes benutzte. Nach ihrer Flucht stürzte sie sich von einer Brücke und erlitt schwere Verletzungen.

**Setting:** Psychiatrische Klinik, geschlossene Station

**Parteien:** Klägerin: Patientin selbst; Beklagte: Krankenhausträger

**Entscheidung:** Die Berufung der Klägerin war erfolgreich. Das Gericht stellte einen schuldhaften Organisationsmangel fest, da keine Anweisung existierte, die Hofaußentür nur unter Absicherung durch eine zweite Person zu benutzen. Der Einwand des Mitverschuldens wurde zurückgewiesen.

### OLG Stuttgart, Urteil vom 20.10.1994 - 14 U 38/93

**Inhalt:** Die anhaltende "Basissuizidalität" eines stationär behandelten, psychisch kranken Patienten rechtfertigt allein noch keine ständige Verwahrung auf einer geschlossenen Abteilung, solange keine krisenhafte Zuspitzung mit erkennbarer akuter Suizidalität vorliegt.

**Sachverhalt:** Der Kläger unternahm einen Suizidversuch während eines ärztlich gestatteten Ausgangs mit seiner Mutter im Rahmen seiner stationären psychiatrischen Behandlung.

**Setting:** Psychiatrische Universitätsklinik (offene psychiatrische Station)

**Verfahrensbeteiligte:** Kläger: Patient; Beklagte: Universitätsklinik und Chefarzt der Klinik

**Entscheidung:** Die Berufung des Klägers hatte keinen Erfolg. Bei dauerhafter Suizidalität müssen gewisse Restrisiken in Kauf genommen werden, um eine angemessene Therapie zu ermöglichen.

### OLG Stuttgart, Beschluss vom 03.02.1997 - 4 Ws 230/96

**Inhalt:** Ein Arzt einer Psychiatrischen Klinik für Kinder und Jugendliche ist verpflichtet, bei der Aufnahme einer Patientin mit Suizidgefahr deren Gepäck zu durchsuchen.

**Sachverhalt:** Eine 17-jährige Patientin hatte unbemerkt einen Kälberstrick mit in die Klinik gebracht und sich damit erhängt (Suizid).

**Setting:** Psychiatrische Klinik für Kinder und Jugendliche (geschlossene Abteilung)

**Verfahrensbeteiligte:** Antragssteller: Eltern der verstorbenen Patientin; Beschuldigter: Ärztlicher Direktor einer Psychiatrischen Klinik für Kinder und Jugendliche. Verfahren im Bereich des Strafrechts (Klageerzwingungsverfahren durch die Eltern der Verstorbenen).

**Entscheidung:** Das Klageerzwingungsverfahren wurde eingestellt, nachdem der Beschuldigte eine Geldbuße nach § 153a StPO gezahlt hatte. Das Gericht bestätigte jedoch das pflichtwidrige Versäumnis der Gepäckdurchsuchung.

### OLG Naumburg, Urteil vom 08.02.2000 - 1 U 140/99

**Inhalt:** Bei der Behandlung suizidgefährdeter Patienten in der Psychiatrie kann die Inkaufnahme von Risiken therapeutisch notwendig sein, wobei dem Arzt ein weiter Beurteilungs- und Ermessensspielraum zukommt. Wurde die Suizidgefahr des Patienten erkannt und hierfür wesentliche Umstände nicht außer Acht gelassen und dieser Gefahr im Rahmen der Therapie entsprechend dem ärztlichen Standard begegnet, scheidet eine Haftung des behandelnden Arztes bzw des Krankenhausträgers aus.

**Sachverhalt:** Suizid einer Patientin durch Fenstersturz.

**Setting:** Psychiatrische Klinik (ohne Angabe, ob offene oder geschlossene Station)

**Verfahrensbeteiligte:** Kläger: Ehemann und Tochter der verstorbenen Patientin; Beklagte: Krankenhausträger

**Entscheidung:** Die Klage wurde abgewiesen, da dem behandelnden Personal ein therapeutischer Entscheidungsspielraum zukommt und keine Pflichtverletzung bei der Einschätzung der Suizidgefahr vorlag.

### BGH, Urteil vom 20.06.2000 - VI ZR 377/99

**Inhalt:** Der Träger einer Psychiatrischen Klinik ist nicht verpflichtet, ohne besondere Umstände alle Fenster einer offenen Station so zu sichern, dass Patienten nicht hinausspringen können.

**Sachverhalt:** Eine Patientin mit schizophrener Psychose stürzte sich nachts vom Balkon eines Aufenthaltsraums der offenen Station im dritten Stock und verletzte sich schwer (Suizidversuch).

**Setting:** Offene Station einer Psychiatrischen Klinik

**Verfahrensbeteiligte:** Klägerin: Patientin; Beklagte: Krankenhausträger

**Entscheidung:** Das Urteil wurde aufgehoben und zurückverwiesen. In einer offenen Station kann ohne besondere Umstände nicht verlangt werden, alle Türen und Fenster verschlossen zu halten.

### OLG Koblenz, Beschluss vom 3.3.2008 - 5 U 1343/07

**Inhalt:** Eine psychiatrische Fachklinik muss suizidalen Patienten den Zugang zu potenziell gefährlichen Gegenständen wie Feuerzeugen verwehren, insbesondere wenn bereits mehrere Suizidversuche unternommen wurden und die Einschränkung das therapeutische Konzept nicht gefährdet.

**Sachverhalt:** Die Patientin hatte vor ihrer stationären Aufnahme bereits zahlreiche Suizidversuche unternommen. Während ihres Klinikaufenthalts kamen zwei weitere Suizidversuche hinzu. Schließlich entzündete die Patientin (Raucherin) in suizidaler Absicht ihre Kleidung mit einem Feuerzeug, was zu schwersten Verbrennungen führte.

**Setting:** Psychiatrische Fachklinik (aus dem Text geht nicht eindeutig hervor, ob offene oder geschlossene Station)

**Parteien:** Kläger: Krankenversicherer der Patientin; Beklagter: Träger der psychiatrischen Fachklinik

**Entscheidung:** Das OLG Koblenz wies die Berufung des Beklagten zurück und bestätigte die Entscheidung des Landgerichts. Die Klinik hatte ihre Sicherungspflichten vernachlässigt, indem sie der suizidgefährdeten Patientin ein eigenes Feuerzeug überließ. Das Gericht erklärte, dass die Gefahr durch einfache Maßnahmen (z.B. Zigaretten nur im Stationszimmer unter Aufsicht anzünden) hätte gebannt werden können, ohne das therapeutische Konzept zu gefährden.

### OLG Frankfurt, Urteil vom 27.10.2009 - 8 U 170/07

**Inhalt:** Ein Allgemeinkrankenhaus ist bei Aufnahme eines akut psychotischen Patienten mit latenter, nicht akuter Suizidgefahr nicht verpflichtet, alle Möglichkeiten einer Selbstschädigung auszuschließen, sondern muss sich intensiv um eine zügige Verlegung in eine psychiatrische Klinik bemühen.

**Sachverhalt:** Eine Patientin mit paranoid-halluzinatorischer Psychose sprang von einem Parkdeck des Krankenhauses und erlitt schwere Verletzungen (Suizidversuch).

**Setting:** Offene Station einer Internistische Station eines Allgemeinkrankenhauses

**Verfahrensbeteiligte:** Klägerin: Patientin; Beklagte: Krankenhausträger

**Entscheidung:** Die Berufung wurde zurückgewiesen, da bei latenter Suizidgefahr nicht jede Selbstschädigungsmöglichkeit ausgeschlossen werden muss und die Beklagte sich ausreichend um eine Verlegung bemüht hatte.

### OLG Zweibrücken, Urteil vom 22.12.2009 - 5 U 5/07

**Inhalt:** Die Pflicht zur Überwachung und Sicherung von suizidgefährdeten Personen in einer psychiatrischen Klinik ist stets im Spannungsfeld zwischen Patientenschutz und Therapiegefährdung zu bewerten und erfordert eine Einzelfallbetrachtung.

**Sachverhalt:** Ein Patient stürzte aus dem Fenster im zweiten Stock einer geschlossenen psychiatrischen Station und verletzte sich schwer (Suizidversuch).

**Setting:** Geschlossene psychiatrische Station

**Verfahrensbeteiligte:** Kläger: Patient; Beklagte: Krankenhausträgerin

**Entscheidung:** Die Berufung des Klägers hatte keinen Erfolg. Die vorhandenen Sicherungsmaßnahmen (abschließbare Fenster mit Sicherungsbügel) waren ausreichend.

### OLG Hamm, Urteil vom 20.01.2010 - 3 U 64/09

**Inhalt:** Eine latent vorhandene Suizidgefährdung bei Patienten einer offenen psychiatrischen Station verlangt nicht, jede Gelegenheit zu einer Selbstschädigung auszuschließen, sondern ist gegen Gesichtspunkte der Therapiegefährdung durch allzu strikte Verwahrung abzuwägen.

**Sachverhalt:** Ein Patient hatte sich mit einem Gürtel an einer Badarmatur erhängt, nachdem es zuvor zu suizidalen Handlungen gekommen war (Suizid).

**Setting:** Offene allgemeinpsychiatrische Station

**Verfahrensbeteiligte:** Kläger: Hinterbliebene (Ehefrau und Söhne); Beklagte: Klinikträger und Chefarzt der Psychiatrischen Klinik

**Entscheidung:** Die Berufung der Kläger wurde zurückgewiesen, da weder ein behandlungsfehlerhaftes Vorgehen noch ein organisatorisches Fehlverhalten feststellbar war.

### OLG München, Urteil vom 13.01.2011 - 1 U 4927/09

**Inhalt:** Bei der Einschätzung einer Suizidgefahr in der Psychiatrie ist maßgeblich, wie sich diese aus der ex-ante-Perspektive des behandelnden Arztes dargestellt hat, wobei eine sichere Suizidprophylaxe bei Wahrung der Menschenwürde und eines zeitgemäßen Therapiekonzeptes nicht möglich ist.

**Sachverhalt:** Ein Patient stürzte sich auf dem Rückweg von einem Termin in der chirurgischen Abteilung ins Treppenhaus und erlitt schwere Verletzungen.

**Setting:** Geschlossene psychiatrische Abteilung

**Verfahrensbeteiligte:** Kläger: Patient; Beklagte: Krankenhausträger, Stationsärztin, Oberarzt

**Entscheidung:** Die Berufung des Klägers wurde zurückgewiesen. Eine sichere Suizidprophylaxe ist bei Wahrung der Menschenwürde und eines zeitgemäßen Therapiekonzeptes nicht möglich.

### LG Gießen, Beschluss vom 28.06.2012 - 7 Qs 63/12

**Inhalt:** Ein Arzt einer Psychiatrischen Klinik macht sich nicht strafbar, wenn er nichts zur Verhinderung eines freiverantwortlich begangenen Selbstmordes unternimmt, auch wenn der betreffende Patient wegen Suizidgefahr überwiesen wurde.

**Sachverhalt:** Ein Patient erhängte sich mit seinem Gürtel im Bad, nachdem die behandelnde Ärztin ihn nicht als suizidgefährdet eingestuft hatte (Suizid).

**Setting:** Station einer Klinik für forensische Psychiatrie (unklar, ob offen oder geschlossen)

**Verfahrensbeteiligte:** Antragsteller: Staatsanwaltschaft; Angeschuldigte: Ärztin einer Psychiatrischen Klinik. Verfahren im Bereich des Strafrechts (Beschwerde gegen den Beschluss zur Nichteröffnung der Hauptverhandlung)

**Entscheidung:** Die Beschwerde wurde zurückgewiesen, da ein eigenverantwortlicher Suizid strafrechtlich nicht dem Arzt zurechenbar ist und dieser sich nicht strafbar macht.

### BGH, Urteil vom 31.10.2013 - III ZR 388/12

**Inhalt:** Der Träger einer Städtischen Klinik ist nicht verpflichtet, sämtliche Fenster einer geschlossenen psychiatrischen Station so zu gestalten, dass sie auch unter Einsatz von Körperkraft von Patienten nicht geöffnet werden können.

**Sachverhalt:** Ein Patient öffnete gewaltsam ein Fenster in seinem Zimmer, sprang aus dem vierten Stock und verletzte sich schwer (Suizidversuch).

**Setting:** Geschlossene psychiatrische Station eines städtischen Klinikums

**Verfahrensbeteiligte:** Kläger: Patient; Beklagte: Stadt als Trägerin des städtischen Klinikums

**Entscheidung:** Die Revision des Klägers wurde zurückgewiesen, da keine Pflicht besteht, alle Fenster gegen gewaltsames Öffnen zu sichern, insbesondere in Räumen, in denen nicht-suizidgefährdete Patienten untergebracht sind.

### LG Münster, Urteil vom 28.10.2021 - 111 O 75/19

**Inhalt:** Bei der Gewährung eines Angehörigenausgangs für einen akut suizidgefährdeten psychiatrischen Patienten muss die begleitende Person über die Suizidgefahr informiert werden.

**Sachverhalt:** Eine Patientin mit akuter Suizidalität erlangte bei einem Angehörigenausgang mit ihrem Vater unter einem Vorwand den Autoschlüssel und beging Suizid im Straßenverkehr.

**Setting:** Geschlossene psychiatrische Station

**Verfahrensbeteiligte:** Kläger: Ehemann der verstorbenen Patientin; Beklagter: Träger der Psychiatrischen Klinik

**Entscheidung:** Der Klage wurde stattgegeben, da der begleitende Angehörige nicht über die akute Suizidgefahr informiert worden war und somit keine angemessenen Vorsichtsmaßnahmen treffen konnte.

### OLG Dresden, Beschluss vom 02.11.2021 - 4 U 1646/21

**Inhalt:** Bei suizidgefährdeten Patienten kann auch die Inkaufnahme des Risikos einer Selbstschädigung therapeutisch geboten sein. Dass der Patient Suizidgedanken äußert, erlaubt daher für sich genommen noch nicht den Schluss auf einen Behandlungsfehler, wenn er im Anschluss hieran einen Suizidversuch unternimmt.

**Sachverhalt:** Ein Patient mit rezidivierenden Depressionen beging nach der von ihm gewünschten Entlassung aus der Psychiatrischen Klinik am selben Abend Suizid.

**Setting:** Psychiatrische Klinik; nicht spezifiziert, ob offene oder geschlossene Station

**Verfahrensbeteiligte:** Kläger: Minderjähriger Sohn des Patienten, vertreten durch die Mutter; Beklagte: Klinikum

**Entscheidung:** Die Berufung des Klägers wurde zurückgewiesen, da der Arzt bei der Entlassung des Patienten sein Ermessen rechtmäßig ausgeübt hatte, der Patient nicht akut suizidal erschien und eine behutsame und schrittweise Lockerung therapeutisch notwendig sein kann.

### OLG Hamm, Urteil vom 20.12.2022 - 26 U 15/22

**Inhalt:** Bei einem akut suizidgefährdeten psychiatrischen Patienten stellt eine Klinikbeurlaubung ohne ausreichende psychiatrische Exploration einen groben Behandlungsfehler dar, wenn diese dem Sicherungsgebot widerspricht.

**Sachverhalt:** Ein 25-jähriger Patient stürzte sich während einer Beurlaubung von einer Brücke und erlitt schwere Verletzungen (Suizidversuch).

**Setting:** Allgemeinpsychiatrische, fakultativ geschlossene Station einer psychiatrischen Klinik

**Verfahrensbeteiligte:** Kläger: Patient; Beklagter: Krankenhausträger

**Entscheidung:** Die Berufung des Beklagten hatte keinen Erfolg. Die Diagnose einer akuten Suizidalität wurde vorschnell aufgegeben und eine Klinikbeurlaubung erfolgte auf unzureichender Befundlage.

### OLG Köln, Urteil vom 17.06.2024 - 5 U 112/23

**Inhalt:** Parasuizidale Handlungen während einer Belastungserprobung eines psychiatrischen Patienten erfordern nach Rückkehr in die Klinik eine ärztliche Untersuchung zur Einschätzung der Suizidalität.

**Sachverhalt:** Ein Patient beging während einer Belastungserprobung Suizid, indem er sich vor eine Straßenbahn stürzte.

**Setting:** Offene Station einer psychiatrischen Klinik

**Verfahrensbeteiligte:** Kläger: Sohn des verstorbenen Patienten; Beklagter: Krankenhausträger

**Entscheidung:** Die Berufung des Klägers wurde zurückgewiesen. Der Kläger konnte nicht beweisen, dass es bei früheren Belastungserprobungen zu parasuizidalen Handlungen gekommen war.

### OLG Köln, Urteil vom 21.08.2024 - I-5 U 127/23

**Inhalt:** Die Gestattung des unbeaufsichtigten Duschens eines akut suizidgefährdeten Patienten auf einer geschlossenen psychiatrischen Station ohne vorherige ärztliche Exploration stellt einen Behandlungsfehler dar, der aber nur bei nachgewiesener Kausalität für den Suizid zu einer Haftung führt.

**Sachverhalt:** Ein Patient erhängte sich mit dem Duschschlauch in der Dusche, nachdem ihm das unbeaufsichtigte Duschen ohne vorherige ärztliche Exploration gestattet worden war.

**Setting:** Geschlossene psychiatrische Akutstation eines Fachkrankenhauses

**Verfahrensbeteiligte:** Kläger: Ehefrau und Söhne des verstorbenen Patienten; Beklagte: Trägerin des psychiatrischen Krankenhauses

**Entscheidung:** Die Berufung wurde zurückgewiesen, da trotz eines möglichen Behandlungsfehlers (Gestattung des unbeaufsichtigten Duschens) die Kläger nicht beweisen konnten, dass dieser Fehler kausal für den Suizid war.

### OLG Hamm, Beschluss vom 03.04.2025 - 5 Ws 48/25

**Inhalt:** Bei einem akut suizidgefährdeten Patienten auf einer geschlossenen psychiatrischen Station stellt das unbeaufsichtigte Duschen keinen Behandlungsfehler dar, wenn sich der Patient hinreichend von der Suizidalität distanziert hat und ein antisuizidales Bündnis geschlossen wurde. Die Abwägung zwischen Sicherungsmaßnahmen und therapeutischen Erfordernissen unter Beachtung der Menschenwürde liegt im Beurteilungsspielraum der behandelnden Ärzte.

**Sachverhalt:** Ein Patient mit paranoider Schizophrenie wurde nach PsychKG auf eine geschlossene Station aufgenommen. Bei der Aufnahme wurde seine Suizidalität als "Hochrisiko" bewertet. Nach einem Gespräch mit dem behandelnden Arzt distanzierte sich der Patient von seiner Suizidalität und schloss ein antisuizidales Bündnis per Handschlag. Am Nachmittag desselben Tages erhängte sich der Patient mit einem Duschschlauch im Badezimmer seines Patientenzimmers (Suizid).

**Setting:** Geschlossene Station einer psychiatrischen Klinik

**Verfahrensbeteiligte:** Antragsteller: Eltern des verstorbenen Patienten; Beschuldigte: Assistenzarzt und Chefarzt der Klinik. Verfahren im Bereich des Strafrechts (Klageerzwingungsverfahren wegen fahrlässiger Tötung).

**Entscheidung:** Der Antrag auf gerichtliche Entscheidung wurde verworfen. Die Ärzte haben die erforderlichen Sorgfaltspflichten nicht verletzt. Das Gericht folgte der gutachterlichen Einschätzung, dass eine den Regeln der ärztlichen Kunst entsprechende Risikoeinschätzung vorlag und ausreichende Überwachungsmaßnahmen eingeleitet wurden. Die Gestattung des unbeaufsichtigten Duschens war unter Berücksichtigung von Menschenwürde, Handlungsfreiheit und therapeutischen Erfordernissen vertretbar.
